# Impact of satellite clinics on geographic access to assisted reproductive technology services in the United States

**DOI:** 10.1186/s12913-022-08281-y

**Published:** 2022-07-19

**Authors:** Micajah Z. McGarity, Christopher N. Herndon, John A. Harris, Benjamin F. Hobbs

**Affiliations:** 1 Department of Environmental Health and Engineering, The Johns Hopkins University, 3400 N. Charles St. Ames Hall 313, Baltimore, MD 21218 USA; 2 Department of Obstetrics & Gynecology, University of Washington, 1959 NE Pacific Street, Seattle, WA 98195-6460 USA; 3 Magee-Womens Research Institute, Pittsburgh, PA USA; 4 Department of Obstetrics, Gynecology and Reproductive Sciences, University of Pittsburgh, 300 Halket Street, Suite 2328, Pittsburgh, PA 15213 USA

**Keywords:** Infertility, Access to care, Applied geography, Assisted reproductive technology, Satellite clinics, Geographic information system

## Abstract

**Background:**

Many assisted reproductive technology (ART) centers utilize satellite clinics to expand reach and access to clinical services, but their contribution to lowering geographic barriers in access to care has not been examined. This study’s purpose is to determine the extent to which satellite clinics impact geographic access to ART and estimate the percentage of reproductive-age women who have geographic access to ART services.

**Methods:**

A systematic web-search collected the locations of all main and satellite ART clinics in the United States (US). Driving times were calculated between satellite clinics and main clinics. The percentage of women with geographic access to care was characterized by clinic type using US Census Core Based Statistical Areas (CBSAs). Logistic regression was used to statistically model the presence of main and satellite clinics as a function of CBSA median income and female reproductive-age population.

**Results:**

Four hundred sixty-nine main clinics with embryology labs and 583 satellite clinics were found in the US. Practices with satellite clinics tend to perform more ART cycles. Satellite clinics are located on average 66 minutes from their practice’s main clinic and 31 minutes from any main clinic. 22% of satellite clinics were in CBSAs without a main clinic. 46 M (72%) US reproductive-age women live in a CBSA with a main clinic, 5.1 M (8%) women live in a CBSA without a main clinic but at least one satellite clinic, and 13 M (20%) women live in an area with no ART clinic of either type. Female reproductive-age population was found to be a more important predictor of clinic presence than median income.

**Conclusions:**

The majority of satellite clinics in the US are positioned in relative proximity to a main clinic. 85% of satellite clinics are located closer to the main clinic of other practices than to their own main clinic. Less than a quarter of ART satellite clinics expand geographic access to ART services by being located in areas without a main clinic, and the vast majority of practices with satellite clinics position their satellite clinics close to another practice’s main clinic.

**Trial registration:**

Not applicable.

## Background

Geographic access to assisted reproductive technology (ART) such as in vitro fertilization (IVF) is a major barrier to entry for millions of infertile women and men throughout the United States (US). ART is one of the most effective and versatile fertility treatments, but it can be prohibitively expensive to those without insurance coverage. It is estimated that only 25% of demand for ART is currently met in the US [1] based on the standard demand estimate of 1500 cycles per million population per year [2]. Barriers to accessing care for many in the US extend beyond cost, and include significant regional geographic barriers in distance to an ART medical center. A study published in 2017 estimated that 28.8% of reproductive-age women in the US do not live in an area with a fertility clinic [3], and a 2010 study using 60-minute driving time to characterize access found 90% of reproductive-age women have geographic access to ART clinics in IVF insurance mandated states and 71% in non-mandated states [4]. These studies examined only ART clinics that have on-site embryology labs (“main clinics”) and did not include clinic sites that do not contain embryology labs on-site and do not generally perform in vitro fertilization (IVF) procedures. These clinics (“satellite clinics”) provide non-ART fertility treatments and also play a critical role in supporting ART cycles through consultations, diagnostic evaluation, bloodwork, and sonographic monitoring.

Geographic access to both main and satellite ART clinics is a crucial dimension of access to ART because ART treatments take place over an extended period of time, on the order of months to years. They also involve numerous office visits, on the order of 10 visits for a single IVF cycle [5]. In aggregate, the time-cost of fertility treatments is considerable. One study found the average time spent on fertility care by 319 couples to be 125 hours, with the majority of the time spent by couples on provider visits (73 hours) [6].

Although satellite clinics are cited as a tool to expand access to care to underserved areas [7], the role that they play in extending geographic access to ART services has not been studied. Location and use of satellite clinics in the US are not reported to or tracked by the US Centers for Disease Control and Prevention (CDC). ART programs are only required to report clinic locations with embryology labs to the CDC as stipulated by the “Fertility Clinic Success Rate and Certification Act of 1992” [8]. The Society for Assisted Reproductive Technology (SART) provides a “Find an IVF Clinic” tool that contains satellite clinic locations for SART member practices [9], but it does not differentiate whether a given clinic is a satellite clinic or a main clinic. Also, SART’s tool does not include non-member fertility clinics, and 20% of clinics in the 2018 reporting to the CDC were not SART members [10].

To the knowledge of the authors, satellite clinic locations have not previously been compiled in a systematic and comprehensive database. As geographic barriers play a significant role in limiting access to care, it is important to better understand the role of satellite clinics. In this study, we provide the first accounting of all satellite clinics nationwide, and use this dataset to conduct a geospatial analysis to understand the geography of satellite clinics and how they impact geographic access to ART.

## Materials and methods

### Systematic web-search design

The approach taken to collect the location of all main and satellite fertility clinics across the US was to perform a systematic web-search of each clinic listed in the CDC’s ART Success Rates dataset from the most recent year available to establish the number and location of all main and satellite clinics. The search was performed in December 2020 and January 2021. The CDC’s 2018 Success Rates dataset [10] was used to identify practices to find online. This systematic search consisted of using the Google search engine to search for the first and second listed names of the practice, the listed city/state, and the name of the medical director. This search method generally resolved the issue that arises when two clinics have similar practice names but different medical directors.

Data collected include the address, contact info, clinic type, and services provided. The dataset was archived with the Johns Hopkins University Data Archive and is publicly available for download [11].

### Geospatial analysis design

To assess the impact of satellite clinics on geographic access to ART, core-based statistical areas (CBSAs) published by the United States Census Bureau were used to define geographic access, similar to a previous study [3]. The 938 CBSAs are defined as metropolitan and micropolitan core areas plus adjacent territory with a “high degree of social and economic integration with the core as measured by commuting ties” [12]. In 2019, the American Community Survey (ACS) 5-year estimate of the total US population was 324,697,795 [13], and the total US population living in CBSAs was estimated at 309,804,779 [14] or 95% of the total US population. With respect to US female reproductive-age population, of the 63,961,819 total women age 20–49 [13], 96% are estimated to live in CBSAs [14]. Additionally, the CDC’s 2018 ART Success Rates Report provides a breakdown of all ART cycles by patient age, with 60% of cycles performed for women younger than 38, and 40% of cycles performed for women age 38 and older [15].

To better understand the factors potentially driving the number of main or satellite clinics, the presence of clinics in a CBSA was modeled as a function of CBSA female reproductive-age population and CBSA median income using logistic regression with standardized covariates. Standard scores, also known as *z*-scores, for reproductive-age population and median income were calculated by first centering by subtracting the mean and then scaling by dividing by the standard deviation. Variable importance was then calculated using the absolute value of the *t*-statistic, computed from the covariate’s regression coefficient divided by its standard error. Lastly, Welch’s independent one tailed *t*-test (assuming unequal variances) was used to compare the number of reproductive-age women and median income between CBSAs with at least one satellite clinic but no main clinic and CBSAs where one or more satellite clinics competes with one or more main clinic.

In addition to exploring the presence of satellite and main clinics within CBSAs, the number of main and satellite clinics was also investigated at the ART practice level. Using the CDC’s 2018 ART-Success Rates dataset, the clinics found in the fertility clinics web search were associated with their corresponding practice using the name of the medical director for the ART practice. For clinics whose names and addresses were matched to the 2018 Success Rates dataset from the CDC, it was assumed that the medical director was the medical director listed in the CDC dataset. Otherwise, the clinic’s medical director was inferred based on information on the clinic’s website or the medical practice’s closest nearby clinic with a matching medical director.

Additionally, the number of clinics per million women aged 20–49 was calculated for each state, and each state was grouped based on if it had an insurance IVF mandate implemented before 2018. The states with insurance mandates for IVF included: AR, CT, HI, IL, MA, MD, NJ, and RI [16]. For main and satellite clinics, the average number of clinics per capita was calculated, and Welch’s independent one tailed *t*-test (assuming unequal variances) was performed, comparing the group means of clinics per million women between states with and without IVF mandates.

Finally, the number of cycles a practice performs was modeled as a function of the number of main and satellite clinics using negative binomial regression, which is typically used to model over-dispersed count response variables [17]. This was appropriate because the variance of the number of cycles a practice performs was found to be higher than the mean.

R 4.0.5 was used for statistical analysis. ArcGIS Pro 2.7.3 (ESRI Corp) was used for GIS analysis and for calculating driving times (regardless of time of day) between main and satellite clinics using a network routing layer from ArcGIS 2019 Business Analyst. The study was determined not to require approval from the Johns Hopkins University Institutional Review Board because we used only publicly available data.

## Results

### Systematic search findings

The systematic-search criteria identified 441 unique practices based in the United States. Together, these practices had a total of 1052 clinic locations, comprising 469 main clinics and 583 satellite clinics in the US.

Of the 441 practices found, 1% (*n* = 5) did not have a main clinic and used another practice’s embryology lab, and in effect operated a satellite clinic at the address listed in the CDC dataset; 93% (*n* = 411) had one main clinic; 5% (*n* = 20) had two main clinics; and 1% (*n* = 5) had three or more main clinics. Most ART programs reported their embryology labs separately and were treated as separate practices, but some programs with multiple embryology labs only have a single record in the CDC Success Rates dataset and were treated as a single practice.

Of the 441 practices found, 50% (*n* = 221) had zero satellite clinics, 18% (*n* = 81) had one satellite clinic, 12% (*n* = 54) had two satellite clinics, 8% (*n* = 36) had three satellite clinics, 4% (*n* = 17) had four satellite clinics, and 7% (*n* = 32) had five or more satellite clinics.

Five clinics associated with practices based in the US were found located abroad. Three satellite clinics were found outside the US, one each in Bermuda, Canada, and Mexico. Two main clinics were located outside the US, one each in Mexico and China. Aside from reporting these findings, facilities located outside the US were excluded from this study.

### Geospatial analysis results

Most satellite clinics were found to be located in proximity to a main clinic. For clinics in the contiguous US, the driving time was calculated between each satellite clinic and its practice’s closest main clinic. The median driving time found was 42 minutes and the mean driving time found was 66 minutes with a 95% confidence interval of 56 to 76 minutes. Practices appear to be placing satellite clinics a good distance away from their main clinics, giving the appearance of expanding geographic access, but most of these satellite clinics are located close to other practices’ main clinics. 85% of satellite clinics are located closer to the main clinic of other practices than to their own main clinic. When calculating the driving time between each satellite clinic and any practice’s closest clinic, the median driving time found was 16 minutes and the mean driving time found was 31 minutes with a 95% confidence interval of 28 to 34 minutes.

**Fig. 1:**
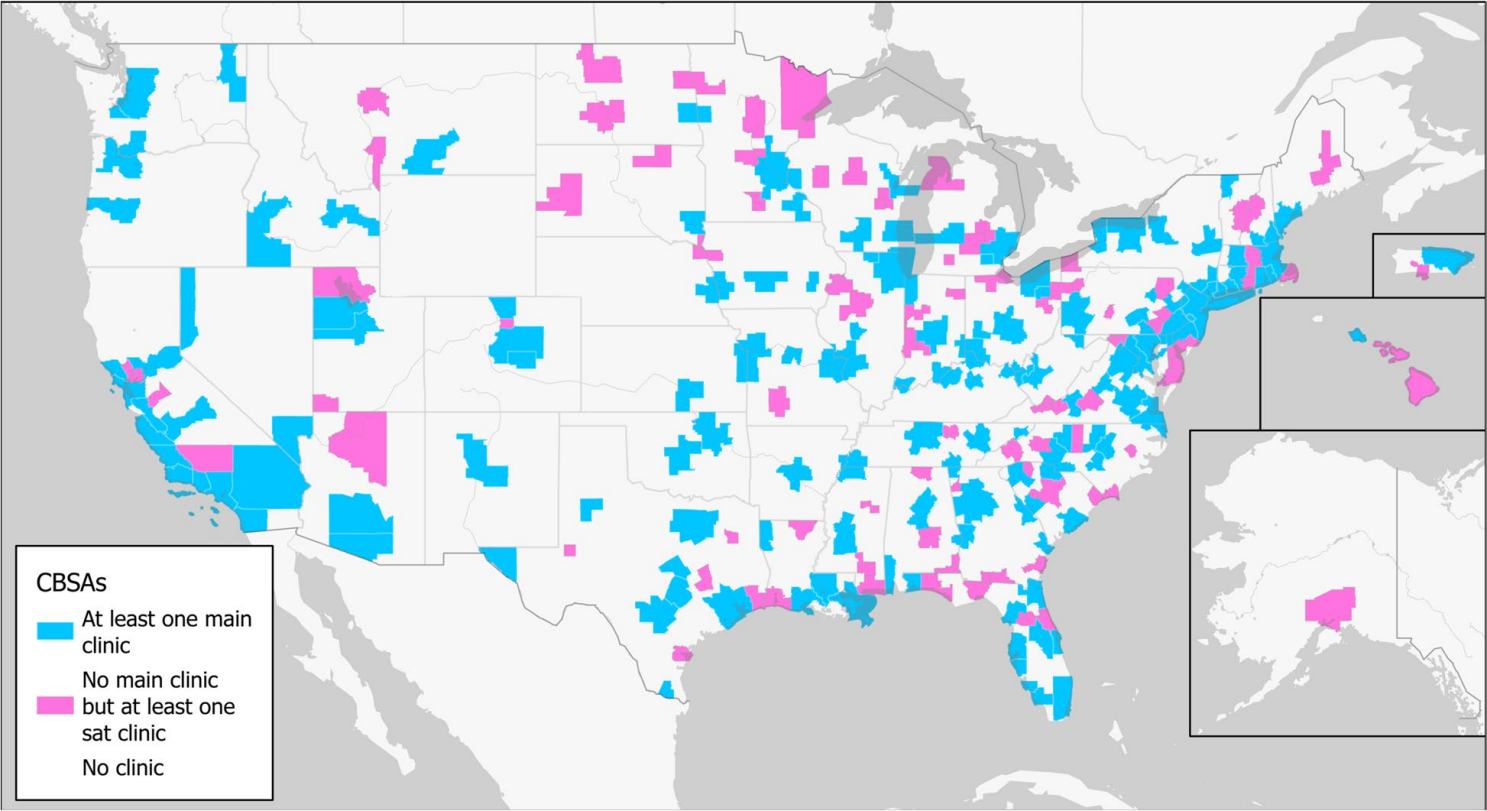
The geographic distribution of main and satellite clinics in the US (including Puerto Rico). CBSAs with at least one main clinic are colored blue, the CBSAs without a main clinic but with at least one satellite clinic in pink, CBSAs without any main or satellite ART clinics in white. Note: all main clinics were located in a CBSA, but three satellite clinics were not located in a CBSA. Basemap used with permission. Source: Esri [18]

Of the 938 CBSAs, 137 CBSAs contained at least one main clinic covering 46 M (72%) US reproductive-age women, 91 CBSAs did not contain a main clinic but contained at least one satellite clinic covering 5.1 M (8%) reproductive-age women, and 710 CBSAs did not contain a clinic of either type, leaving 13 M (20%) reproductive-age women uncovered. Further, 78% (*n* = 451) of satellite clinics were found in CBSAs that also have a main clinic, and 22% (*n* = 129) of satellite clinics were found in CBSAs without a main clinic. All main clinics were located within a CBSA, but there were three satellite clinics not located within a CBSA. A map depicting these findings is shown in Fig. 1. Statistically significant (*p* < 0.001) differences in means for both median income and reproductive-age women were found between CBSAs with a satellite clinic but no main clinic and CBSAs with both a main and satellite clinic, with $11 k higher median income and 423 k more reproductive-age women in CBSAs where satellite clinics compete with main clinics.

**Fig. 2:**
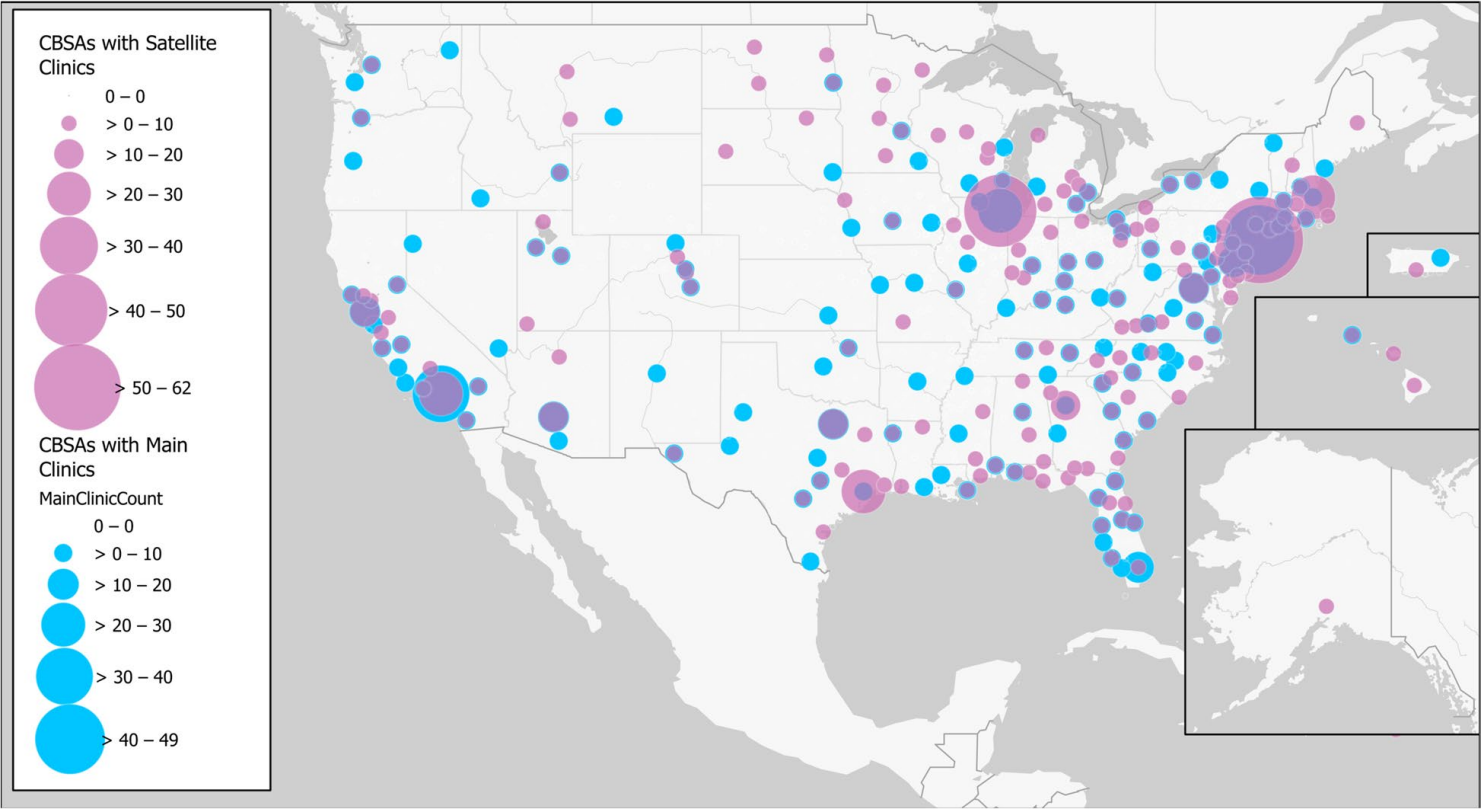
Geographic distribution of fertility clinics sized by the number of main clinics in blue and satellite clinics in pink. Areas with both main and satellite clinics appear purple due to transparency of the satellite clinic layer. Basemap used with permission. Source: Esri [18]

Figure 2 illustrates the concentration and geographic distribution of clinics by CBSA for main clinics and satellite clinics, respectively. Both main clinics and satellite clinics are located across the US. However, the CBSAs with the highest concentration of clinics are generally positioned in urban cores.

Logistic regression with standardized covariates was used to model the presence of both satellite and main clinics in a CBSA. The modeling found both female reproductive-age population and median income to have coefficients with significance values *p* < 0.001. For both clinic types, population was found to be a more important predictor of clinic presence. For main clinics, population was about 3 times more important than median income (based on covariate *t-*statistics), and for satellite clinics, population was about 2 times more important than median income. The results of this modeling are shown in Table 1. Additionally, an increase in one standard deviation in female reproductive age population increases the odds of a clinic being located in a CBSA by 27,000 for main clinics and 66 for satellite clinics. An increase in one standard deviation in median income increases the odds of a clinic being in a CBSA by 1.8 for main clinics and 1.6 for satellite clinics.

**Table 1:** Logistic regression odds ratios exploring the relationship between population and median income on the likelihood of a main or satellite clinic’s presence in a CBSA

	Main Clinic OR [95% CI]	Sat Clinic OR [95%CI]
Intercept	0.19 [0.13–0.28]	0.26 [0.21–0.33]
Female Pop 20–49 Z-Score	27,000 [5000–185,000]	66 [27–173]
Median Income Z-Score	1.8 [1.3–2.5]	1.6 [1.3–2.0]

The number of clinics with respect to US regions was also studied. Regionally, the number of main clinics per practice was 1.1 in the North, South, and Midwest, and 1.0 in the West and Puerto Rico. There was greater variation in the number of satellite clinics per practice regionally. The rate of satellite clinics per practice (ordered from most to least) was 1.8 in the Northeast, 1.5 in the Midwest, 1.3 in the South, 1.0 in the West, and 0.3 in Puerto Rico.

The impact of a state’s IVF insurance mandate on the number of main and satellite clinics per million reproductive age women was also examined. A significant difference was found for satellite clinics, with a difference of means of six more satellite clinics per million women (*p* = 0.04) in insurance mandate states. For main clinics, however, this comparison found a non-significant difference in means of two more main clinics per million women (*p* = 0.18).

At the practice level, the negative binomial regression modeling found a statistically significant (*p* < 0.001) relationship with a positive correlation between a practice’s number of satellite clinics and the number of cycles it performs. There was not a statistically significant (*p* = 0.26) relationship between a practice’s number of main clinics and the number of cycles performed.

## Discussion

This study has several findings relevant to understanding the role that ART satellite clinics currently play in the United States to extend access to ART services. One is that higher volume ART practices are more likely to establish satellite clinics. Secondly, and most importantly, the majority (78%) of satellite clinics do not increase geographic access to care because they are located in a CBSA that also has a main clinic. However, our study indicated there are 129 satellite clinics located in CBSAs without a main clinic, thereby extending geographic access to care to 8% of the US reproductive-age female population (5.1 million women).

The analysis of driving times between satellite and main clinics suggests that most satellite clinics are placed by practices in order to compete for patients, rather than to increase geographic access to underserved populations. Satellite clinics are located on average 66 minutes away from *their* practice’s main clinic but only 31 minutes away from *any* practice’s main clinic. These driving times suggest that the primary roles of satellite clinics are to improve patient convenience and increase market share. The tendency for competing businesses to locate near each other is described in Hotelling’s Law in economics that states that competing sellers will tend to locate close to each other to obtain the largest market share, and “as more and more sellers of the same commodity arise, the tendency is not to become distributed in the socially optimum manner but to cluster unduly” [19]. The findings that 78% of satellite clinics are placed in CBSAs and that CBSAs in these areas have on average $11 k higher median income suggest that satellite clinics are placed primarily to compete for patients in proven markets, consistent with Hotelling’s Law. Considering that travel time was found to be the second largest contributor to the substantial time-cost of fertility care [6] and that patients are willing accept a clinic with a lower pregnancy rate in exchange for reductions in travel time [20], the satellite clinic placement strategy we have identified is likely effective in competing for patients with long travel times within their CBSA, but less effective at reaching patients in unserved CBSAs who could potentially gain access to ART through a local satellite clinic.

At the practice level, the analysis supports the hypothesis that satellite clinics allow practices to reach more patients. Looking at the roughly half of all practices that operate satellite clinics, those practices tend to perform more cycles than practices lacking satellite clinics. For example, only one practice with zero satellite clinics performed more than 2000 cycles, whereas 25 practices with at least one satellite clinic performed more than 2000 cycles. Exploring these trends further, the negative binomial regression modeling estimated one satellite clinic for practices performing 400 cycles, two for 1700 cycles, three for 2500 cycles, four for 3000 cycles, and five for 3500 cycles. Based on this trend, practices operating fewer satellite clinics than their number of cycles indicate should consider opening a new satellite clinic. Conversely, practices operating more satellite clinics than their number of cycles would indicate should consider if there are better locations to operate their satellite clinics that could reach more patients.

Economic access to ART treatments is a primary barrier to care in the United States. The majority of insurance plans do not include coverage for fertility treatments because only a fraction of states have IVF insurance mandates [16]. These “IVF mandates” have been shown to increase utilization rates [21], but they do not apply to all insurance plans held in each state. In 2020, of the 91.4% of Americans with health insurance, 52.9% obtained it through their employer [22]. Because of federal law [23], IVF mandates do not apply to the estimated 50–60% of workers with health plans self-insured by their employer [24], so it is unclear how many employers provide IVF coverage, but a 2015 survey of 462 mainly large, private employers found 27% included coverage for IVF [25]. In 2021, the federal government did not offer insurance plans with ART coverage to its employees [26], and Americans who obtain health insurance through the federal government do not receive coverage for ART, except for wounded veterans [27] and Medicaid recipients in Utah with genetic disorders [28]. Therefore, most patients in the US are required to pay out-of-pocket for ART treatments. Practices that desire to select an economically viable location to place a new clinic should consider potential patient base with respect to population as well as the population’s ability to afford services based on income.

ART practices likely operate under a profit-maximization framework, as is common in both for-profit (a majority of ART practices) and not-for-profit healthcare organizations [29, 30]. Currently unserved, highly-populated areas were identified that are likely to yield profitable ART practices. Assuming that existing practice locations are profitable, the current approach to siting clinics may be the best guide for locating new clinics with the aim of incrementally increasing geographic access to care while boosting the chance of new clinics staying in business. Therefore, with the aim of finding new locations for successful ART clinics, the logistic regression model predicting the presence of main clinics was applied to find 5 CBSAs that the model predicts are most likely to have a main clinic, but actually have no clinic of either type at present (shown in Table 2). Positioning new clinics in these CBSAs would extend access to ART to about a half a million women in total. The logistic regression models indicate that these currently unserved areas are likely promising areas to locate a new main or satellite clinic.

**Table 2:** Top five CBSAs without an ART main or satellite clinic ranked by logistic regression-modeled likelihood of a main clinic

CBSA	Female Pop. Age 20–49	Median Household Income ($)	Likelihood Main Clinic	Likelihood Satellite Clinic
Stockton, CA Metro Area	146,788	64,432	93%	64%
Lakeland-Winter Haven, FL Metro Area	126,869	50,584	72%	42%
Fayetteville-Springdale-Rogers, AR Metro Area	107,239	57,603	61%	40%
Salem, OR Metro Area	80,727	60,178	35%	31%
Visalia, CA Metro Area	90,163	49,687	32%	26%

State IVF insurance mandates may have an impact in certain areas and influence the establishment of satellite clinics. Our analysis comparing state-level data found on average significantly more satellite clinics per million women in IVF mandate states. Future studies should explore the effect of IVF mandates on geographic access to care, particularly in context of the recent increase in number of states adopting mandates for insurance coverage of IVF.

The findings of this research are likely significant to the ART community as a whole, which seeks to reduce barriers and disparities in access to ART based on the creation of the American Society for Reproductive Medicine’s Access to Care Initiative in 2015 [7]. Additionally, this study’s findings would be useful to ART practices located near specific areas identified as currently unserved but likely to support a new clinic. These nearby practices to these identified areas could use this study’s findings as justification for expanding operations via opening new clinics or partnering with local OB/GYN practices to bring ART monitoring to underserved populations and expand their potential pool of patients. Finally, state and local policy makers seeking to improve access to care could apply these findings to craft subsidies to draw new ART clinics to underserved areas.

### Limitations

The web-search relied on information publicly available on practice websites. This may not reflect the true state of clinics due to out-of-date or incorrect website information. Additionally, several ART practices have partnerships with other non-ART OB/GYN practices to provide ultrasound and monitoring support for IVF cycles. If the partnership was detailed on an ART practice’s website, then the non-ART OB/GYN practice was considered a satellite clinic of the ART practice. If the partnership was omitted from the ART practice’s website, it was not captured in this study.

This study did not investigate the level of care provided by each satellite clinic and treated all satellite clinics as equivalent, although services provided at satellite clinics vary. For instance, some satellite facilities are open one day per week for consultation only, while others provide monitoring for ART and non-ART fertility treatments 7-days per week.

This study investigated only if the *existence* of state IVF insurance mandates had an impact on the number of ART clinics per capita and did not investigate specific aspects of IVF mandates. IVF mandates were treated as equivalent, but there are substantial differences in IVF mandates between states with respect to coverage, eligibility criteria, and maximum benefit. Future studies should investigate the specifics of IVF mandates and their potential impact on geographic access to ART.

Finally, the use of US Census CBSAs to define geographic access to care is an approximation of actual geographic access. The geographic area defining a CBSA is selected based on high levels of social and economic integration measured by commuting times [12]. However, long travel times within large CBSAs may inhibit access to care for some patients. Also, patients living outside of a CBSA with a fertility clinic but within a reasonable travel time to the clinic would not be counted as having access. Lastly, some patients may be willing to travel outside of their home CBSA for care.

## Conclusions

Satellite clinics in aggregate extend geographic access to ART to millions of women, but most individually do not because they are often located in proximity to a main clinic. Less than a quarter of all satellite clinics extend geographic access to CBSAs lacking a main clinic. Our study identified many areas in the US where unmet need exists that could potentially support the presence of a new clinic and would help expand geographic access to care to large underserved populations.

## Data Availability

The datasets generated during and/or analyzed during the current study are available in the Johns Hopkins Data Archive at 10.7281/T1/XZ0CGJ and 10.7281/T1/QXKY4B

## Abbreviations

ART: Assisted reproductive technology
CDC: US Centers for Disease Control and Prevention
CBSA: US Census Core Based Statistical Areas
IVF: In vitro fertilization
OB/GYN: Obstetrics and gynecology
SART: The Society for Assisted Reproductive Technology
US: The United States of America
